# Towards an integrative hope-dysfunctional beliefs perspective to personal recovery in schizophrenia: a path analysis

**DOI:** 10.1186/s12888-023-05135-7

**Published:** 2023-09-04

**Authors:** Daniel Fu Keung Wong, Yves Cho Ho Cheung, Xiaoyu Zhuang, Yat-nan Petrus Ng, Lindsey G. Oades, Shengquan Sam Ye

**Affiliations:** 1 Department of Social Work, Baptist University of Hong Kong, AAB1035, 10/F, Academic and Administration Building, 15 Baptist University Road, Baptist University Road Campus, Kowloon Tong, Hong Kong; 2 Sociology Research Center, School of Humanities, Jinan University, 601, Huangpu Avenue West, Tianhe District, Guangzhou City, Guangdong Province China; 3 Melbourne Graduate School of Education, The University of Melbourne, Melbourne Graduate School of Education, The University of Melbourne, Grattan Street, Parkville, VIC 3010 Australia; 4 Department of Social and Behavioural Sciences, City University of Hong Kong, Department of Social and Behavioural Sciences, City University of Hong Kong, Tat Chee Avenue, Kowloon, Hong Kong

**Keywords:** Schizophrenia, Recovery, Negative symptoms, Defeatist beliefs, Asocial beliefs, Strength-based, Cognitive-behavioral therapy

## Abstract

**Background:**

Evidence shows that negative symptoms of schizophrenia and underlying dysfunctional cognition are related to persistently low functioning and quality of life. However, despite the abundance of existing recovery programs for people with schizophrenia, few have examined whether and how the widely-adopted hope-motivation recovery pathway and the deficit-oriented cognitive pathway might converge to influence functioning and quality of life.

**Methods:**

A cross-sectional, quantative survey recruited a convenient sample of adult outpatients with DSM-5 schizophrenia spectrum disorders and low social functioning (*n* = *124*). Self-reported measurements included personal recovery (30-item Mental Health Recovery Measure), social functioning (8-item Social Functioning Questionnaire), hope (12-item Hope Scale), quality of life (28-item World Health Organization Quality of Life Scale-Abbreviated Version-Hong Kong), defeatist beliefs (15-item extracted from Dysfunctional Attitude Scale), and asocial beliefs (15-item extracted from Revised Social Anhedonia Scale). Correlation analysis and structural equation modelling was applied to investigate how the two pathways intertwined to predict social functioning and quality of life.

**Results:**

Asocial beliefs and hope separately mediated two partial mediation pathways from defeatist beliefs to recovery outcomes (social functioning and personal recovery). Meanwhile, defeatist beliefs, social functioning, and personal recovery further predicted quality of life.

**Conclusions:**

This is one of the very few studies that provides empirical evidence of a deficit-strength linkage in the recovery from schizophrenia. Remediation of dysfunctional beliefs and the injection of hope and successful experiences should be undertaken concurrently in recovery as they are associated with differential effects on enhancing social functioning and personal recovery, which then converge and contribute to a better quality of life.

## Background

According to the World Health Organization [1] schizophrenia affects around 0.32% of the population and 0.45% of adults globally. As a severe mental illness, schizophrenia can drastically hinder a person’s daily functioning [2] and quality of life [3]. Evidence shows that negative symptoms of schizophrenia, such as anhedonia and amotivation, are associated with lower functioning [4] and poorer quality of life [5] among people with schizophrenia and are more medication-resistant than positive symptoms [6]. In addition, Grant et al. [7, 8] have found that cognitive deficits such as defeatist and asocial beliefs held by people with schizophrenia are adversely affecting the functioning and quality of life of this group of people. On the other hand, empirical studies have suggested that inducing hope and building strength in people with schizophrenia could also facilitate their better social functioning and quality of life. However, it is surprising to note from the existing literature that there is a lack of empirical studies that examine the connection between a cognitive deficit model and a strength-based model in affecting the social functioning and quality of life of people with schizophrenia. Investigating this connection is of vital importance for both theoretical and practical reasons. Theoretically, both therapeutic pathways exist in reality and influence a person with severe mental illness. However, there were few attempts to synthesize the two pathways into a coherent model of understanding the factors affecting negative symptoms and poor social functioning among people with severe mental illness. Practically, clarity about this connection will provide insight into developing an integrated intervention model that facilitates recovery of people with severe mental illness.

Existing literature reports two major lines of enquiry concerning the occurrence and maintenance of negative symptoms and social functioning of people with schizophrenia. One involves a defeatist-asocial beliefs pathway derived from the work of Beck et al. [9]. Essentially, they identified two types of cognition, defeatist beliefs (also called defeatist performance beliefs) and asocial beliefs associated with worsening negative symptoms and poorer social functioning of people with severe mental illness. Defeatist beliefs are self-defeating beliefs related to goal-directed tasks (e.g., “*taking even a small risk is foolish because the loss is likely to be a disaster*”) ([10]^(p. 67)^), and asocial beliefs concern aversive social attitudes and self-isolating beliefs (e.g., “*I could be happy living all alone on my own*”) ([7]^(p. 70)^). These beliefs can interact and impede motivation, hamper activity engagement, and limit people’s opportunity to generate positive experiences [9]. Indeed, evidence shows that defeatist beliefs are associated with amotivation and less effortful goal-pursuing behavior [8]. Some studies suggested that neurocognitive and social-cognitive impairments may underline the development of defeatist beliefs and the consequential effects on negatives symptoms and social functioning [11]. However, other studies have found that defeatist beliefs, feelings of stigmatization and negative expectations can result in amotivation and a lack of effortful goal-directed behaviors, leading to poor cognitive and behavioral task performance. In turn, this poor performance may generate poor self-esteem and self-efficacy among people with severe mental illness and further reinforce their self-defeatist behaviors [12]. Meanwhile, people experiencing self-defeating beliefs and social and internalized stigma may more readily embrace asocial beliefs leading to lower social and community participation and social withdrawal [13].

This line of analysis has generated the application of recovery-oriented cognitive therapy (CT-R) to neutralize negative beliefs and attitudes while activating the people’s underlying positive attitudes and interests to promote adaptive living [14]. In essence, the worker facilitates a person to identify personal and meaningful goals (e.g., reconnecting with family) and to achieve the goals gradually. Through this process, the worker helps the person to process the successful experiences to shift the their mindset from defeatist and asocial beliefs to embracing success and social and community integration [9]. Indeed, clinical interventions using CT-R found improvement in global functioning and negative symptoms compared with controls, especially among people with more chronic schizophrenia. Specifically, successful experiences in daily living were associated with higher self-esteem, lower defeatist beliefs and better mood [15]. People undergoing CT-R became more reengaged, had more energy and motivation, and were more open to talking about future aspirations. In addition, two studies on cognitive-behavioral social skills training (CBSST) found defeatist and asocial beliefs mediated the effect of treatment on negative symptoms and functioning [16].

However, some studies have reported mixed or not-better-than-control effects on dysfunctional beliefs and social withdrawal at post-intervention or follow-up [13]. Another issue is that while the linkage between defeatist beliefs and negative symptoms and social functioning appears to be established, the linkage between asocial beliefs and social functioning remains unclear [17]. There is still a lack of clarity about the pathway on the interrelationship between defeatist beliefs, asocial beliefs, negative symptoms and social functioning.

The second line of enquiry regarding the occurrence and maintenance of negative symptoms and social functioning of people with severe mental illness involves a hope-motivation-strength pathway that is derived from a strength-based recovery approach to mental illness. According to Slade et al. [18], recovery is an ongoing process of personal growth, healing, and self-determination. It is essentially strength-focused and emphasizes a person’s capacity to identify and develop their internal strengths and external resources that promote hope and a meaningful life of the people. Andresen et al. [19] have proposed a five-stage model of recovery that includes.

*“(a) moratorium: a time of withdrawal characterized by a profound sense of loss and hopelessness; (b) awareness: realization that all is not lost and that a fulfilling life is possible; (c) preparation: taking stock of strengths and weaknesses regarding recovery and starting to work on developing recovery skills; (d) rebuilding: actively working toward a positive identity, setting meaningful goals, and taking control of one’s life; and (e) growth: living a meaningful life characterized by self-management of illness, resilience, and a positive sense of self*” ([19]^(p. 976)^).

Others describe recovery from mental illness as a journey from a passive, disengaging, withdrawing, and disparaging self to one characterized by a sense of hope, optimism, and meaning and purpose in life [20]. The journey itself is nonlinear and complex and does not mean that people recovering from mental illness do not experience any psychiatric symptoms, have no struggles, and can be completely independent in meeting all their needs. The recovery-oriented, strength-based approach has become an important component in the delivery of mental health care in different parts of the world [18]. It represents an articulation of the philosophy of recovery. It aims to facilitate people with mental illness to develop personal goals and aspirations and to identify and secure a range of environmental and personal resources for developing a life full of meaning and purpose [21]. Essentially, this approach to mental health care signifies a shift of primary focus from illness and deficits to strength and personal growth of a personwith mental illness. Two recent meta-analyses have examined the effectiveness of the recovery-oriented strengths-based approach for people with severe mental illness. The meta-analysis of Ibrahim et al. [22] examined five studies reporting that the strength-based approach was not superior to other service delivery models. The meta-analysis done by Tse et al. [23] included seven studies highlighted the effectiveness of the strength-based approach in improving employment, educational, and intrapersonal outcomes.

Although many scholars have suggested the importance of hope and strength in facilitating the recovery of people with severe mental illness [24, 25], the hope-motivation-strength pathway is not clearly or adequately supported by empirical intervention studies [22]. Moreover, the theoretical frameworks articulated by different scholars have implied rather than empirically examined the connection between the hope-motivation-strength pathway and the defeatist-asocial pathway.

### Research objectives

The current study aimed to examine how the above two pathways are meaningfully and empirically connected. First, it examined the relationship between hope, personal recovery, social functioning, and quality of life. Second, it further investigated how defeatist beliefs and asocial beliefs were associated with hope, personal recovery, social functioning, and quality of life. Third, It examined the pathways from defeatist beliefs and asocial beliefs to quality of life, mediated by hope, personal recovery, and social functioning.

### Hypotheses

**Fig. 1:**
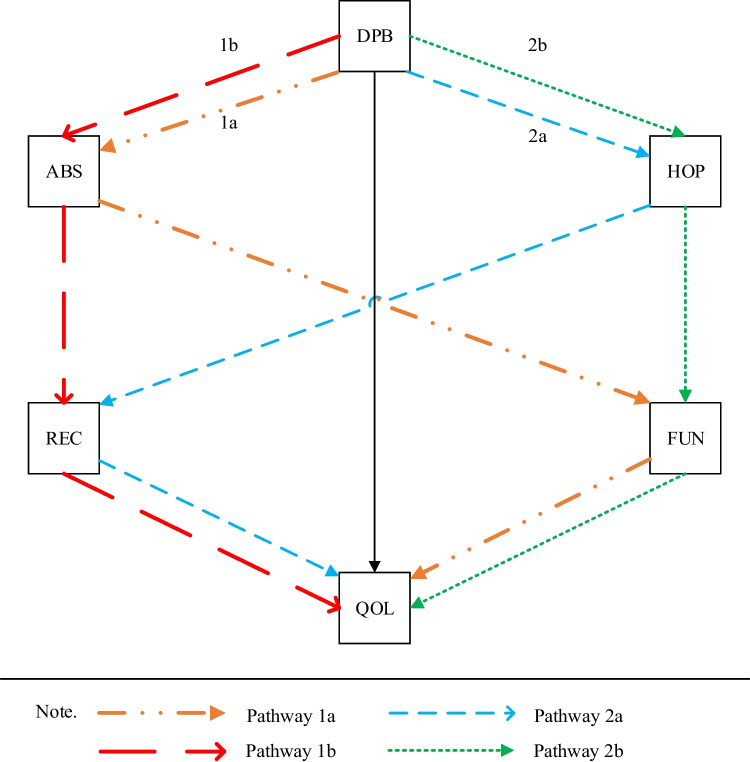
Hypothesized model. DPB = Defeatist beliefs, ABS = Asocial beliefs. HOP = Hope. FUN = Social functioning. REC = Personal recovery. QOL = Quality of life

First, we made hypotheses regarding correlations. H1: hope, personal recovery, social functioning, and quality of life would be positively intercorrelated. H2: defeatist beliefs and asocial beliefs would negatively correlate with the four constructs in H1. We further hypothesized the four pathways illustrated in Fig. 1. Pathway 1a: asocial beliefs, and subsequently social functioning, would mediate the effect of defeatist beliefs on quality of life. Pathway 1b: asocial beliefs, and subsequently personal recovery, would mediate the effect of defeatist beliefs on quality of life. Pathway 2a: hope, and subsequently personal recovery, would mediate the effect of defeatist beliefs on quality of life. Pathway 2b: hope, and subsequently social functioning, would mediate the effect of defeatist beliefs on quality of life.

## Methods

### Procedures and informants

A cross-sectional study was conducted by recruiting a convenience sample of 126 informants at six government-sponsored community mental health centers in Hong Kong from July 2020 to June 2021. Inclusion criteria were (a) a diagnosis of DSM-5 schizophrenia spectrum disorders [26], (b) experiencing poor social functioning during the screening period, as indicated by a score of at least 10 in the Social Functioning Questionnaire [27] at the time of recruitment, (c) compliant to medications, (d) aged 18 to 65, (e) able to understand Cantonese or Chinese. Exclusion criteria were (a) neurological disease or damage that would compromise cognitive functioning, (b) physical handicap that would interfere with assessment procedures, or (c) suicidal attempt or ideation in the past three months.

### Instruments

A printed questionnaire containing the following scales was administrated to the informant after written consent was obtained. All data collected were self-reported. Each informant received HKD50 (~ USD6.4) upon questionnaire completion.

#### Personal recovery

The 30-item Mental Health Recovery Measure [28] assesses informants’ self-perceived personal recovery on a 5-point Likert scale. A higher score indicates a higher level of recovery. The Chinese version was validated by Ye et al. [29]. Internal consistency was excellent in the current study, *Cronbach’s α* = *0.95*, with subscale *α* ranging between *0.68* and *0.90.*

#### Social functioning

The 8-item Social Functioning Questionnaire [27] is a self-reported survey equivalent to the Social Functioning Schedule interview. It assesses eight aspects of social functioning: occupation, home tasks, finance, relationships, sexual life, social activities, isolation, and spare time activities on a 4-point Likert scale. A higher score indicates poorer social functioning. The Chinese version used by Wang et al. [30] reached an acceptable level of internal consistency. However, internal consistency in the current study was poor, *Cronbach’s α* = *0.55*.

#### Hope

The 12-item Hope Scale developed by Snyder et al. [31] was administered. It contains two subscales (agency and pathways) using an 8-point Likert scale. A higher score indicates being more hopeful. The Chinese version has been used by Ho et al. [32]. Internal consistency in the current study was excellent, *Cronbach’s α* = *0.90*.

#### Quality of life

The 28-item World Health Organization Quality of Life Scale-Abbreviated Version-Hong Kong [33] has been translated and validated in Chinese by Leung et al. [34]. It contains four domains (physical, psychological, social, and environmental), with two additional items on general quality of life and health. It is scored on a 5-point Likert scale and converted into scores ranging from 0 to 100. A higher score indicates a higher quality of life. Domain-specific internal consistency in the current study was questionable to good, *Cronbach’s α*_*physical*_ = *0.75, α*_*psychological*_ = *0.81, α*_*social*_ = *0.60, α*_*environmental*_ = *0.82.*

#### Defeatist beliefs

Following the method of Grant and Beck [9] and Granholm et al. [35], the 15-item Defeatist Performance Attitude subscale was extracted from the Dysfunctional Attitude Scale [10]. It is scored using a 7-point Likert scale, and the Chinese version was validated by Wong et al. [36]. A higher score indicates having lower defeatist beliefs. Internal consistency in the current study was good, *Cronbach’s α* = *0.88*.

#### Asocial beliefs

Following the method of Grant and Beck [9] and Granholm et al. [35], a subset comprising 15 items was extracted from the Revised Social Anhedonia Scale [37]. It is scored using a 2-point Likert scale and a higher score indicates having higher social distancing beliefs. The Chinese version has been used by Chan et al. [38] with good internal consistency. Internal consistency in the current study was acceptable, *Cronbach’s α* = *0.69*.

### Data analysis

Descriptive and correlation analyses were performed using IBM SPSS Statistics version 26. Two informants who had omitted responses to more than 5% of the questionnaire were excluded from the analysis (final *n* = *124*). Multiple imputations were applied to missing data which was missing completely at random (Little’s MCAR test, *χ*^*2*^*(2526)* = *2417.765, p* = *0.94*). Then, descriptive analyses were conducted on demographic and measured variables, followed by correlation analyses to identify associations (Pearson's r) between variables.

Structural equation modeling analysis was conducted with R 4.2.2 [39] with package “lavaan” [40]. First, to simplify our model, a latent construct “quality of life” was created to explain covariance between four domains of quality of life, with the model fit tested under a confirmatory factor analysis (CFA; R function “cfa”). Second, a path analysis (R function “sem”) was conducted to examine the overall model fit. Model fit criteria of the CFA and path analysis include chi square (χ^2^), comparative fit index (CFI), Tucker Lewis Index (TLI), standardized root mean square residual (SRMR), and root mean square error of approximation (RMSEA). Third, bootstrapping analysis with 5,000 resamples were performed to determine the bias-corrected 95% confidence intervals [41] of each mediation paths. To minimize the possibility of Type I error in marginal situations, the significance of the mediation paths was further checked with the test of joint significance, in which the mediation was deemed significant if all individual paths constituting the compound path were significant [42].

## Results

### Demographics

Of the 124 informants, 58.9% were female. Their age ranged between 19 and 81 years, with a mean (SD) of 42.70 (13.10). Regarding education, 3.2% of informants graduated from the 6^th^ grade, 34.7% graduated from the 9^th^ grade, 37.1% graduated from high school, and 25.0% graduated from tertiary education. Regarding medication, 96.0% had regular medication prescribed by a doctor, 0.8% had no medication, and 3.2% had unstable medication. Meanwhile, 93.5% received periodic case management follow-up, and 6.5% received irregular follow-up. In the previous six months, informants had been hospitalized for a mean (SD) of 2.09 (8.72) days and 0.12 (0.39) times.

### Means, SDs and cutoffs

Means, standard deviations (SD) and correlations are shown in Table 1. The Mean (SD) of social functioning was 10.84 (3.24), with 65.3% of informants scoring equal to or higher than the cut-off score of 10, indicating poor social functioning [27].

**Table 1:** Means, standardized deviations (SDs) and correlations of variables

			Correlations							
	Mean	SD	Social Functioning	Personal Recovery	Hope	QOL Physical	QOL Psychological	QOL Social	QOL Environmental	Defeatist Beliefs
Social Functioning	10.84	3.24								
Personal Recovery	75.04	17.74	-.47***							
Hope	39.48	10.90	-.39***	.70***						
QOL Physical	51.56	16.67	-.49***	.54***	.49***					
QOL Psychological	44.49	18.42	-.46***	.69***	.72***	.64***				
QOL Social	43.69	18.23	-.41***	.51***	.40***	.50***	.58***			
QOL Environmental	50.08	16.88	-.42***	.55***	.41***	.54***	.61***	.58***		
Defeatist Beliefs	59.52	15.20	-.20*	.13	.22*	.29**	.35***	.33***	.31***	
Asocial Beliefs	6.48	3.12	.37***	-.25**	-.14	-.28**	-.22*	-.20*	-.24**	-.43***

*Note*. *QOL* Quality of Life

*p < .05, **p < .01, ***p < .001

### Correlations

Higher hope, better personal recovery, better functional recovery, and better quality of life in four domains were all associated (all *p* < *0.05*). Besides, having higher defeatist beliefs was associated with poorer social functioning (*r* = *-0.20, p* < *0.05*), lower hope (*r* = *0.22, p* < *0.05*), poorer quality of life (*r*_physical_ = *0.29, p* < *0.01, r*_psychological_ = *0.35, p* < *0.001, r*_social_ = *0.33, p* < *0.001, r*_environmental_ = *0.31, p* < *0.001*), and higher asocial beliefs (*r* = *-0.43, p* < *0.001*). However, defeatist beliefs were not correlated with personal recovery. Meanwhile, higher asocial beliefs were associated with poorer social functioning (*r* = *0.37, p* < *0.001*), poorer personal recovery (*r* = *-0.25, p* < *0.01*), poorer quality of life (*r*_physical_ = *-0.28, p* < *0.01, r*_psychological_ = *-0.22, p* < *0.05, r*_social_ = *-0.20, p* < *0.05, r*_environmental_ = *-0.24, p* < *0.01*), although asocial beliefs were not correlated with hope.

### Structural equation modeling

The confirmatory factor analysis (CFA) of quality of life showed an excellent fit (*χ*^*2*^ = *2.41, p* = *0.30, CFI* = *1.00, TLI* = *0.99, RMSEA* = *0.04, 90%CI [0.00, 0.19], SRMR* = *0.02*).

**Fig. 2:**
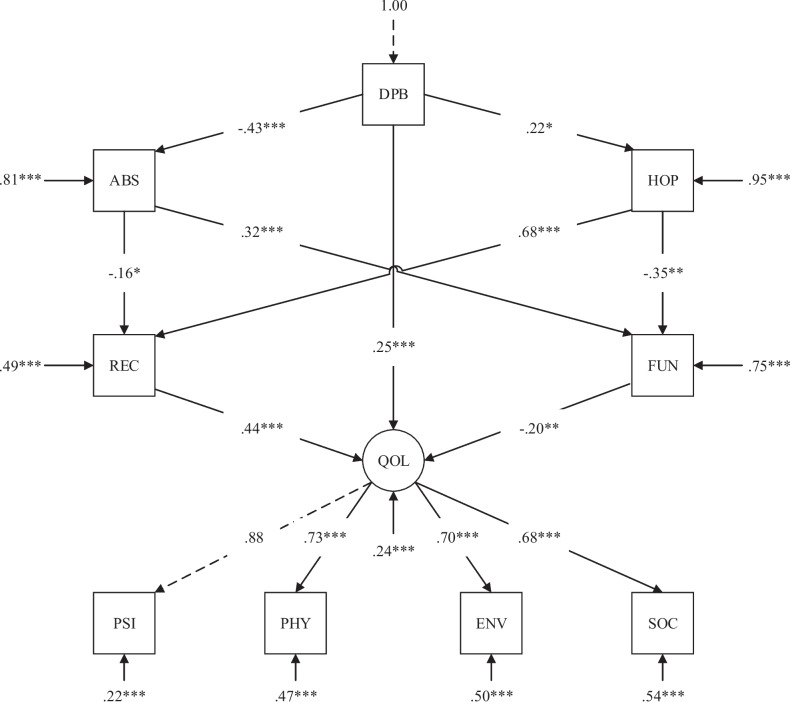
SEM model. *Note*. Dashed lines represented fixed parameters. DPB = Defeatist beliefs, ABS = Asocial beliefs. HOP = Hope. FUN = Social functioning. REC = Personal recovery. QOL = Quality of life. PSI = Psychological domain. PHY = Physical domain. ENV = Environmental domain. SOC = Social domain. * *p* < 0.05, ** *p* < 0.01, *** *p* < 0.001

Figure 2 presents the path diagram, and Table 2 the result of bootstrapping. Model fit was good (*χ*^*2*^ = *41.07, p* < *0.01, CFI* = *0.96, TLI* = *0.94, RMSEA* = *0.08, 90%CI [0.04, 0.12], SRMR* = *0.05*), and the whole model explained 75.7% of the variance of the latent factor “quality of life”. Mediation pathways 1a (*β* = *0.03, p* < *0.05, 95% CI [0.01, 0.06]*) and pathway 1b (*β* = *0.03, p* < *0.05, 95% CI [0.00, 0.07]*) were significant, whereas pathway 2a (*β* = *0.07, p* < *0.10, 95% CI [0.01, 0.16]*) and pathway 2b (*β* = *0.02, p* < *0.10, 95% CI [0.00, 0.04]*) were marginally significant. Although some confidence intervals included zero when rounded, none of the lower boundaries fell into negative values, and the test of joint significance [42] supported the significance of all four specified paths.

**Table 2:** Four mediation pathways from defeatist beliefs to quality of life under 5,000 bootstrapping

	Beta	Z-value	95% CI
Pathway 1a: Defeatist beliefs ➔ Asocial beliefs ➔ Social functioning ➔ Quality of life	.03	2.20*	[.01, .06]
Pathway 1b: Defeatist beliefs ➔ Asocial beliefs ➔ Personal recovery ➔ Quality of life	.03	2.02*	[.00, .07]
Pathway 2a: Defeatist beliefs ➔ Hope ➔ Personal recovery ➔ Quality of life	.07	1.89†	[.01, .16]
Pathway 2b: Defeatist beliefs ➔ Hope ➔ Social functioning ➔ Quality of life	.02	1.70†	[.00, .04]
Direct effect: Defeatist beliefs ➔ Quality of life	.25	4.04***	[.14, .40]
Total effect	.39	5.04***	[.26, .58]

*Note*. †*p* < .10, *p < .05, **p < .01, ***p < .001

## Discussion

This study examined the interwining pathways to recovery among people with severe mental illness. While the results confirm some current understanding, they also uncover some interesting pathways that have not been expirically explored in the literature. Indeed, some of the newly found pathways bridge the cognitive-behavioral model [14] and the hope-recovery model [18] commonly found in the current literature.

### Defeatist-asocial beliefs pathways

We hypothesized that defeatist beliefs, via asocial beliefs, influence the social functioning and sense of recovery of a person recovering from severe mental illness. The results of path analysis indicated that fewer defeatist beliefs were associated with fewer asocial beliefs, and in turn, predicted higher social functioning (pathway 1a). Interestingly, fewer defeatist beliefs and asocial beliefs were also found to be associated with a greater sense of personal recovery (pathway 1b). These two pathways ultimiately converged into better quality of life for the person. Both findings are consistent with existing literature [10, 14] and seem to suggest that despite suffering from severe mental illness, those with fewer defeatist beliefs may have a more positive outlook on life and be more willing to participate in social and functional activities, leading to better social functioning. Similarly, a person with severe mental illness with a more positive outlook on life will have a better sense of personal recovery (e.g., better positive attitudes towards mental illness and greater hope toward recovery), resulting in better quality of life as a whole. The findings echo a defeatist-asocial belief pathway and reaffirm the importance of helping people with severe mental illness develop strategies to modify their defeatist and asocial beliefs and enhance their overall positive attitudes in life by engaging in activities that provide them with successful experiences and help them build up confidence and motivation to continue to engage in recovery-oriented activities. The Cognitive Therapy-Recover Model initiated by Grant et al. [14] exemplifies this approach to improving the lives of people with severe mental illness.

### Hope-motivation-strength pathway

Supporting our hypotheses, this study also confirms the hope, motivation and strength pathway towards better social functioning and quality of life for people with severe mental illness (Pathway 2a and 2b). Research has repeatedly found hope an indispensable factor underpinning the process of personal recovery [20]. In essence, hope represents “the beliefs that it is possible for someone to regain a meaningful life, despite serious mental illness” ([24]^(p. S621)^), motivating people to make the change (Pathway 2b). In the hope-personal recovery pathway (Pathway 2a), however, a person does not only embrace a general positive outlook in life, but they also take action to identify and utilize internal and external resources (i.e., strengths) to work towards achieving life goals that enhance their meaning in life. Indeed, the current recovery movement engineered by scholars such as Slade et al. [18] and Rapp et al. [20] has adopted this line of enquiry to improve the lives of people with severe mental illness.

### Asocial beliefs-social functioning and asocial beliefs-personal recovery pathways

Our results in pathways 1a and 1b revealed that fewer asocial beliefs predicted higher social functioning and higher personal recovery. To the best of our knowledge, this is one of very few studies that has established two relationships stemming from asocial beliefs to social functioning and personal recovery respectively, particularly among an Asian population. Previous research had hypothesized but had not fully, empirically substantiated such possible relationships. One of the thorny issues facing by people with severe mental illness is social withdrawal, which has a strong link to deterioration in social functioning and poor mental health [43]. Our current empirical findings not only affirm the need to develop strategies to enhance the social connectedness of people with severe mental illness but also highlight the importance of fostering positive attitudes and beliefs towards the need for interpersonal relationships among people with severe mental illness. Indeed, recovery from severe mental illness is very much an interpersonal process that requires continuous support from peers, family members, friends, colleagues, religious groups, and community member [44]. These provide people with connectedness and a sense of belonging, opportunities for social learning and practicing skills, instrumental and emotional support, positive feedback and encouragement [45]. Thus, while it is essential to facilitate a person with severe mental illness to be linked to others socially, it is equally important to help them to process the connection in a meaningful way so that they are able to appreciate the benefits through such connection.

### Defeatist beliefs and hope pathway

As shown by the results, hope mediated the effect of defeatist beliefs on personal recovery and social functioning. The pathway from defeatist beliefs to hope is another new and interesting result in our study. Indeed, this is one of the few studies providing empirical support for the linkage between defeatist beliefs and hope, thus bridging the two dominant lines of enquiry on recovery for people with severe mental illness. This linkage is not difficult to understand given that people with severe mental illness, such as schizophrenia, have neurocognitive deficits in memory and attention. These deficits contribute to possible unsuccessful goal attainment in life, which over time, can give rise to dysfunctional, defeatist attitudes about oneself and one’s performance. These dysfunctional attitudes, in turn, may lead to dissatisfaction and a sense of despair and hopelessness. On the other hand, a reduction in defeatist beliefs can increase the sense of hope among people with severe mental illness and then develop into different hope pathways identifed by our model.

### An integrative hope-dysfunctional beliefs recovery approach

This is one of the first few studies that have attempted to examine the connection between a cognitive deficit and hope-strength connection in the recovery of people with schizhophrenia. The conventional hope pathway in mental health recovery programs would suggest that an increase in hope, say, through building successful experiences, can build a strength-oriented momentum, mitigate negative presumptions and enhance a person’s motivation to make further positive changes [44]. But our findings highlight the significance of defeatist beliefs as underlying factors influencing hope and asocial beliefs, which further lead to poor social functioning and quality of life of people with severe mental illness. It adds another dimension, on top of existing recovery model, to suggest that cognitively changing one’s defeatist beliefs through different means can induce a sense of hope and lead a person to the hope pathway. This provides a diversion to the conventional recovery programs, which claim that irrespective of deficits, the focus on strength building can independently enhance the full recovery of a person. Our study suggests, in line with the cognitive model of negative symptoms postulated by Beck et al. [9], perhaps, the need for a more balanced deficit-and-strength perspective to conceptualize the lives of people with severe mental illness and interventions. While it is important to explore and facilitate a person to use their internal strength and external resources to achieve full recovery, there is also a need to work through various deficits to maximize optimal recovery in a person. Thus, a future recovery-oriented approach should pay attention to both deficits and strengths of the people in both assessment and interventions.

### Limitations

The current study had several limitations. First, the data were collected through a cross-sectional survey. Although path analysis can test our theoretically constructed model against the data, our result could not ascertain the temporal, causal relationship between variables. Further studies with longitudinal design and clinical trials are required to further establish the causality among variables. Second, all the measurements were based on self-reported scales, which could be affected by subjective factors. Some studies have reported that self-reported quality of life and functioning could be affected by insight [46]. Further studies may adopt multi-rater, role-play (e.g., for social functioning) [17], or clinician-reported approaches to mitigate potential bias in self-reported data. Third, the internal consistency of the social functioning questionnaire was poor. We conducted further analyses and found that the low inter-item correlations were not caused by any single item in the scale. In our data, inter-item correlations of the social functioning scale were generally mediocre. Since the eight items in the scale measured perceived functioning in eight aspects of life, it might indicate that, for people with schizophrenia, high functioning in one aspect of life is not associated with high functioning in another aspect. Fourth, the current study did not divide informants into deficit vs. nondeficit groups. Some scholars have suggested deficit and non-deficit schizophrenia are two distinct disorders [47]. Hence, further studies may investigate how heterogeneity within diagnosis or the existence of positive symptoms may affect therapeutic effectiveness. Fifth, there could be a lack of referenced literature from the last five years. Meanwhile, referenced studies in this study were conducted in the last decade, after the publication of cognitive theory of negative symptoms by Grant and Back in 2009 – 2010 [7–9]. Further studies will be required to enlighten researchers on the association between cognition, negative symptoms, and recovery.

## Conclusions

This study provides empirical support of an integrated multiple pathway model to full recovery for people with severe mental illness. It answered the research objectives that, first, hope, personal recovery, social functioning, and quality of life were positively correlated. Second, higher defeatist beliefs was associated with poorer social functioning, lower hope, poorer quality of life, and higher asocial beliefs; whereas higher asocial beliefs were associated with poorer social functioning, poorer personal recovery, and poorer quality of life. Third, it also empirically illustrates that the defeatist-asocial cognition pathway and the hope-motivation-strength pathway stem from defeatist beliefs, intertwine and converge into quality of life. On the one hand, defeatist beliefs predicted asocial beliefs, which predicted quality of life as mediated by personal recovery and social functioning; on the other hand, defeatist beliefs also predicted hope, which further predicted quality of life, mediated by personal recovery and social functioning.

The findings emphasize the importance of attending to both strengths and deficits when assessing and working with people with severe mental illness. While it is important to continue to uphold the hope-motivation-strength pathway to recovery, it is also important to pay attention to the defeatist-asocial pathway as these can demoralize people in the recovery process. Indeed, when designing intervention programs for people with severe mental illness, both aspects should be considered to provide a more balanced recovery approach to them.

## Data Availability

The anonymous dataset analyzed during the current study are available from the corresponding author on reasonable request.

## Abbreviations

CT-R: Recovery-Oriented Cognitive Therapy
CBSST: Cognitive-behavioral Social Skills Training
DSM-5: Diagnostic and Statistical Manual of Mental Disorders, Fifth Edition
CFA: Confirmatory Factor Analysis
CFI: Comparative Fit Index
TLI: Tucker Lewis Index
SRMR: Standardized Root Mean Square Residual
RMSEA: Root Mean Square Error of Approximation
